# Efficacy, safety, and transcriptomic mechanisms of Fuzheng Yangxin formula for enhanced recovery after coronary artery bypass grafting: a propensity score-matched prospective cohort study

**DOI:** 10.3389/fmed.2026.1902379

**Published:** 2026-08-12

**Authors:** Jinyang Yu, Yifei Wang, Yunhu Li, Tong Ding, Luyu Meng, Xiaochang Ma, Yunpeng Ling, Yichen Gong

**Affiliations:** 1 Department of Cardiac Surgery, Peking University Third Hospital, Beijing, China; 2 State Key Laboratory of Natural and Biomimetic Drugs, Peking University, Beijing, China; 3 Department of Cardiovascular Disease, Xiyuan Hospital, China Academy of Chinese Medical Sciences, Beijing, China; 4 Graduate School, Beijing University of Chinese Medicine, Beijing, China; 5 Department of Traditional Chinese Medicine, Peking University Third Hospital, Beijing, China; 6 National Clinical Research Center for Chinese Medicine Cardiology, Xiyuan Hospital, China Academy of Chinese Medical Sciences, Beijing, China; 7 State Key Laboratory of Traditional Chinese Medicine Syndrome, Xiyuan Hospital, China Academy of Chinese Medical Sciences, Beijing, China

**Keywords:** coronary artery bypass grafting, enhanced recovery after surgery (ERAS), Fuzheng Yangxin formula, immune microenvironment, propensity score matching, transcriptome sequencing

## Abstract

**Background:**

This study aimed to evaluate the clinical efficacy, safety, and transcriptomic mechanisms of Fuzheng Yangxin Formula (FZYX) for enhanced recovery after coronary artery bypass grafting (CABG) in patients with Qi-Yin deficiency syndrome.

**Methods:**

This prospective cohort study utilized 1:1 propensity score matching (PSM). Matched patients (*n* = 144; 72/group) received a 7-day FZYX intervention plus conventional therapy or conventional therapy alone. Assessments included the Seattle Angina Questionnaire Physical Limitation (SAQ-PL) score at 7 days, peak serum creatinine (Cr), B-type natriuretic peptide (BNP), and perioperative complications. Transcriptomic mechanisms were explored via peripheral blood RNA sequencing.

**Results:**

Post-intervention, the FZYX group demonstrated significantly lower peak Cr (68.50 vs. 74.50 μmol/L, unadjusted *p* = 0.010; adjusted *p* = 0.0059 via ANCOVA) and peak BNP (751.00 vs. 1231.50 pg/mL, *p* = 0.019). Although the difference in absolute SAQ-PL scores did not reach statistical significance for superiority (40.00 vs. 35.56, *p* = 0.055), the FZYX group exhibited a markedly reduced quartile coefficient of dispersion (11.1% vs. 20.0%), indicating a trend towards a more stable physical recovery.

**Conclusion:**

FZYX appears to be a safe and promising adjunctive therapy post-CABG. While the primary endpoint was formally negative, exploratory analyses suggest it may provide target organ protection and stable physical recovery, potentially associated with modulating macrophage/monocyte-driven innate inflammation, promoting adaptive immune reconstitution, and accelerating tissue repair.

**Clinical trial registration:**

Identifier NCT06961136, https://clinicaltrials.gov/study/NCT06961136

## 1 Introduction

Coronary artery bypass grafting (CABG) remains the gold standard for the treatment of severe and complex coronary artery disease (1). Although continuous optimizations in surgical techniques and perioperative management in recent years have mitigated macroscopic trauma to some extent (2), myocardial ischemia–reperfusion injury, systemic micro-inflammatory stress, reactive oxygen species (ROS) accumulation, and local tissue damage induced by the surgical procedure itself remain inevitable (3–5). These pathophysiological alterations significantly hinder patients’ early postoperative recovery. In the early post-CABG phase, patients frequently face severe physical limitations, target organ stress (such as acute kidney injury and ventricular overload), and often fall into a state of “immune paralysis” that makes them highly susceptible to infections (6, 7). Therefore, exploring novel enhanced recovery after surgery (ERAS) strategies that can effectively resolve excessive inflammatory responses, restore systemic immune homeostasis, and actively accelerate tissue repair has become a critical and urgent clinical need in the field of cardiovascular surgery.

In recent years, the significant potential of Traditional Chinese Medicine (TCM) in the treatment of cardiovascular diseases has gained widespread recognition from the international medical community (8). Multiple large-scale randomized controlled trials published in top-tier medical journals, such as Nature Medicine and the Journal of the American Medical Association (JAMA), have provided solid evidence-based medical support for the efficacy of TCM formulas in ameliorating heart failure and reducing adverse cardiovascular events (9, 10). However, the application of TCM in the acute recovery phase following cardiac surgery remains largely underexplored. Current conventional Western medical interventions post-surgery predominantly focus on the secondary prevention of coronary artery disease (e.g., antiplatelet therapy, lipid lowering), lacking targeted interventions capable of modulating the systemic “immune-inflammatory” network and actively promoting wound and tissue healing (11, 12).

Based on traditional TCM theory, patients undergoing CABG experience major surgical trauma and severe fluid depletion, with the core pathogenesis predominantly manifesting as “Qi-Yin deficiency syndrome.” To address this, our team developed the Fuzheng Yangxin Formula (FZYX), primarily consisting of the root of *Panax ginseng* C.A.Mey. (Araliaceae), the processed lateral root of *Aconitum carmichaelii* Debeaux (Ranunculaceae), the root of *Astragalus membranaceus* (Fisch.) Bunge (Fabaceae), the root of *Ophiopogon japonicus* (Thunb.) Ker Gawl. (Asparagaceae), the root of *Angelica sinensis* (Oliv.) Diels (Apiaceae), the root of *Rehmannia glutinosa* (Gaertn.) DC. (Orobanchaceae), the root and rhizome of *Rhodiola crenulata* (Hook.f. & Thomson) H. Ohba (Crassulaceae), and the root powder of *Panax notoginseng* (Burkill) F. H. Chen (Araliaceae). This formula aligns with the traditional therapeutic principles of “replenishing Qi and nourishing Yin, promoting blood circulation, and removing blood stasis” (13). Our previous network pharmacology and *in vivo* studies verified that FZYX effectively treats cardiovascular conditions associated with these syndromes by protecting cardiomyocytes, inhibiting apoptosis, and improving overall cardiac function (14, 15). The pharmacological profiles of its primary active compounds directly address the core requirements of postoperative recovery:

*Remodeling of immune homeostasis*: Studies have shown that Ginseng extracts (e.g., Ginsenoside Rg1) exert potent anti-inflammatory effects by regulating the polarization balance of M1/M2 macrophages and gut microbiota homeostasis (16). Salidroside, a core natural compound in Rhodiola, has similarly been identified as a potential therapeutic target for modulating M1/M2 macrophage polarization (17). This precise regulation of macrophages is crucial for attenuating the excessive innate immune response post-surgery.

*Promotion of wound and tissue healing*: Astragalus extracts have been proven to act synergistically with mesenchymal stem cells (MSCs), mediating immune modulation via paracrine signaling to significantly accelerate wound healing (18). Furthermore, preconditioning with salidroside can accelerate the repair of complex surgical wounds by enhancing the paracrine function of MSCs and improving their survival rate in harsh microenvironments such as ischemia or hyperglycemia (19).

*Comprehensive synergistic effects*: In the formula, Danggui and Sanqi powder synergistically improve microcirculation and endothelial function (20); Maidong and Shengdihuang maintain cellular metabolic homeostasis (21); and Fupian warms Yang to support Heart Qi (22). Active components such as ginsenosides and salidroside may exert protective effects through robust antioxidant capacity and inflammatory regulation. The multi-target, multi-pathway synergistic effects endow FZYX with the pharmacological potential to “inhibit excessive injury, rebuild immune defense, and initiate tissue repair.”

To fill the clinical gap regarding the application of TCM in postoperative recovery after cardiac surgery, we conducted a prospective observational cohort study. We enrolled CABG patients postoperatively diagnosed with Qi-Yin deficiency syndrome and applied a rigorous propensity score matching (PSM) method to systematically evaluate the effects of FZYX (administered consecutively for 7 days) as an adjunct to conventional therapy on patients’ early quality of life and target organ function. Furthermore, by integrating peripheral blood whole-transcriptome sequencing (RNA-seq) with single-sample gene set enrichment analysis (ssGSEA), this study aims to elucidate the precise mechanisms underlying the multi-dimensional biological effects (especially immune remodeling and tissue repair) of FZYX in postoperative recovery at the molecular and cellular network levels, thereby providing robust translational evidence for the integration of TCM-Western medicine strategies into the ERAS system for cardiovascular surgery.

## 2 Materials and methods

### 2.1 Study design and ethical approval

This study was a single-center, prospective observational cohort study (ClinicalTrials.gov identifier: NCT06961136). All research procedures strictly adhered to the ethical principles of the Declaration of Helsinki and were officially approved by the Medical Science Research Ethics Committee of Peking University Third Hospital (Ethics Approval No.: IRB00006761-M2024555). All patients included in this study were fully informed of the study objectives and potential risks prior to participation and provided written informed consent.

### 2.2 Study population, inclusion/exclusion criteria and sample size estimation

This study consecutively screened patients with coronary artery disease who underwent isolated off-pump minimally invasive CABG at our center.

The inclusion criteria were as follows: (1) a definitive diagnosis of coronary atherosclerotic heart disease and undergoing CABG surgery; (2) a postoperative Traditional Chinese Medicine (TCM) diagnosis of Qi-Yin deficiency syndrome. The diagnosis of “Qi-Yin deficiency syndrome” was established on postoperative day 1 by professional TCM physicians. To ensure diagnostic reproducibility and inter-observer consistency, the syndrome classification was independently cross-verified by two senior TCM practitioners. This diagnosis was made in accordance with validated TCM syndrome differentiation criteria for cardiovascular diseases reported in recent peer-reviewed literature (13), based on patients’ primary symptoms (chest pain, chest tightness), secondary symptoms (palpitations, shortness of breath, fatigue, dry mouth, sweating), and tongue and pulse conditions.

The main exclusion criteria included: (1) allergy to the components of the FZYX granules; (2) concomitant severe hepatic or renal dysfunction; (3) acute infection and fever postoperatively; (4) concurrent valvular or other intracardiac surgeries; (5) end-stage malignancy or inability to tolerate regular follow-up.

The clinical sample size calculation was primarily driven by the superiority hypothesis for the primary endpoint (SAQ Physical Limitation score at 7 days postoperatively). Based on preliminary trials and previous literature, the expected mean SAQ score in the control group was approximately 55.56 with a standard deviation of 17. We hypothesized an expected mean difference of approximately 9.5 points between the two groups. Setting the strict superiority margin at 1 point (i.e., the lower boundary of the 95% confidence interval for the difference must be greater than 1), with a one-sided alpha level of 0.025 and a statistical power of 80%, a minimum of 65 patients per group was required. Accounting for an estimated 15% dropout or loss to follow-up rate, the target enrollment was approximately 85 patients per group. To facilitate rigorous 1:1 propensity score matching (PSM) and adequately adjust for baseline confounders, this prospective cohort planned to initially enroll 100 patients in the FZYX group and 200 in the control group. This enrollment ratio ensured that the final matched cohort would maintain sufficient statistical power for the superiority analysis.

For the transcriptomic analysis, standard RNA-seq guidelines recommend a minimum of three biological replicates per group. We employed stratified random sampling prior to PSM, initially collecting 30 peripheral blood samples on postoperative day 3 (20 from the control group and 10 from the FZYX group, aligning with the initial 2:1 cohort ratio). Following the clinical PSM procedure, sequencing samples from patients excluded during matching were removed to maintain baseline consistency. Ultimately, 18 matched peripheral blood samples (10 in the control group and 8 in the FZYX group) were included for RNA extraction and downstream sequencing analysis.

### 2.3 Plant material and FZYX granules preparation

The FZYX intervention utilized single-herb extract granules manufactured by Beijing Chunfeng Traditional Chinese Medicine Co., Ltd., China, in strict accordance with Good Manufacturing Practice (GMP) standards and the 2015 edition of the Chinese Pharmacopoeia (batch number 20250429). All plant species mentioned in this manuscript have been cited with their full botanical names, including authorities, and verified against the World Flora Online (WFO) Plant List (https://wfoplantlist.org/; accessed on 1 May 2025). The procurement and processing of these botanical materials by the manufacturer complied with relevant national biodiversity regulations and guidelines.

The daily formula is composed of single-herb granules equivalent to the following raw herb amounts: 20 g of the root of *Panax ginseng* C. A. Mey. (Araliaceae), 10 g of the processed lateral root of *Aconitum carmichaelii* Debeaux (Ranunculaceae; decocted first), 30 g of the root of *Astragalus membranaceus* (Fisch.) Bunge (Fabaceae), 20 g of the root of *Ophiopogon japonicus* (Thunb.) Ker Gawl. (Asparagaceae), 15 g of the root of *Angelica sinensis* (Oliv.) Diels (Apiaceae), 15 g of the root of *Rehmannia glutinosa* (Gaertn.) DC. (Orobanchaceae), 30 g of the root and rhizome of *Rhodiola crenulata* (Hook.f. & Thomson) H. Ohba (Crassulaceae), and 6 g of the root powder of *Panax notoginseng* (Burkill) F. H. Chen (Araliaceae; taken dissolved in water). All individual granules were compounded and dispensed by the central pharmacy of Peking University Third Hospital. To ensure intervention quality and consistency, the comprehensive chemical profile of the FZYX granules was characterized via ultra-performance liquid chromatography coupled with quadrupole time-of-flight mass spectrometry (UPLC-Q-TOF-MS) (Supplementary Figure 1), which successfully identified 129 principal constituents—predominantly saponins, flavonoids, phenylpropanoids, and alkaloids—with detailed mass spectral data provided in Supplementary Table 1.

### 2.4 Treatment protocol and clinical data collection

Patients were assigned to either the experimental group (FZYX group) or the control group based on their willingness to receive TCM intervention in addition to conventional therapy. Both groups received standardized conventional Western medical therapy post-CABG. On this basis, the experimental group began oral administration of compounded FZYX granules on postoperative day 1 for 7 consecutive days (5.4 g of compounded granules per dose, three times daily). Clinical follow-up and baseline data were prospectively collected and double-verified using the Electronic Data Capture (EDC) system (REDCap) provided by the Peking University Clinical Research Institute, ensuring data authenticity and traceability.

### 2.5 Clinical outcome measures

Primary outcome measure: The Physical Limitation (PL) dimension score of the Seattle Angina Questionnaire (SAQ) after 7 days of intervention, used to accurately assess patients’ early postoperative quality of life and physical recovery status.

Secondary outcome measures: These included perioperative organ stress and safety evaluations, specifically including peak postoperative serum creatinine (Peak Cr), peak B-type natriuretic peptide (Peak BNP), intensive care unit (ICU) stay duration, mechanical ventilation time, total postoperative hospital stay, as well as the incidence of major adverse cardiovascular and cerebrovascular events (MACCE, including myocardial infarction, stroke, repeat revascularization, or all-cause mortality) and other major complications (e.g., poor wound healing, new-onset atrial fibrillation, renal failure requiring dialysis).

The diagnosis of major complications, including postoperative myocardial infarction, was defined according to the Fourth Universal Definition of Myocardial Infarction (Type 5 MI). All clinical endpoint events were evaluated and adjudicated by an independent committee of two senior cardiologists who were blinded to the treatment allocation.

### 2.6 Peripheral blood RNA sequencing and differentially expressed gene analysis

To explore the potential molecular mechanisms of the drug’s efficacy, we collected peripheral blood samples on postoperative day 3 of the intervention and extracted total RNA for whole-transcriptome sequencing (RNA-seq). Oligo(dT) magnetic beads were used to enrich mRNA from total RNA, which was then fragmented and synthesized into double-stranded cDNA using random hexamer primers. Library construction was subsequently completed through end repair, A-tailing, sequencing adapter ligation, size selection, and PCR amplification. After the libraries passed quality control utilizing Qubit quantification and fragment distribution analysis via a Bioanalyzer, sequencing was performed on the Illumina platform (based on the sequencing-by-synthesis principle).

The raw reads generated from the sequencer were first subjected to strict quality control using fastp software to remove adapter-containing, poly-N, and low-quality reads, yielding high-quality clean reads, with Q20, Q30, and GC content statistics calculated. Subsequently, the paired-end clean reads were efficiently mapped to the human reference genome using HISAT2 (version 2.2.1) software. The generated sequencing count matrix, after filtering (retaining genes with standard deviation > 0), underwent logarithmic transformation [Log_2_(x + 1)] and principal component analysis (PCA) to evaluate the consistency of overall expression profiles among samples between groups. The identification of differentially expressed genes (DEGs) was performed using the DESeq2 package based on the negative binomial distribution model. To ensure the selection of targets with definitive biological significance, the significance thresholds were strictly set at an adjusted *p*-value (padj) < 0.05 and an absolute log_2_ fold change (|log_2_FC|) > 0.5.

### 2.7 Gene set enrichment analysis (GSEA) and single-sample gene set enrichment analysis (ssGSEA)

To elucidate the biological functions of the drug intervention from a global network perspective, we performed Gene Set Enrichment Analysis (GSEA) utilizing the clusterProfiler package and the org.Hs.eg.db database. The analysis was based on a ranked list of log2FoldChange values for all sequenced genes and enriched using the Biological Process (BP) ontology. To preserve biological signals reflecting the multi-target synergistic micro-regulations of the TCM formula, which often elude strict multiple testing corrections (e.g., FDR) in highly heterogeneous clinical cohorts, the threshold was set at a Nominal *p*-value < 0.05 for exploratory hypothesis generation. To strictly control for false positives and ensure biological plausibility, a rigorous manual curation filter was applied to the nominal results to exclude noise pathways completely unrelated to cardiovascular surgery (e.g., reproduction, hair follicles), focusing exclusively on pathways pertinent to CABG prognosis. For the evaluation of the immune microenvironment, we utilized single-sample gene set enrichment analysis (ssGSEA, implemented via the GSVA package, with minSize set to 1) to quantify the relative abundance of 28 immune cell subpopulations in peripheral blood. Finally, the Spearman rank correlation coefficient was used to calculate the intrinsic associations between key DEGs and the infiltration abundances of the 28 immune cells.

### 2.8 Statistical analysis

Continuous variables were first assessed for normality using the Shapiro–Wilk test. Non-normally distributed continuous variables, including the primary endpoint (SAQ scores) and postoperative laboratory markers, were expressed as medians with interquartile ranges (Median [IQR]) and compared between groups using the Wilcoxon rank-sum test. Additionally, to evaluate the predefined superiority of the primary endpoint, the mean difference and its two-sided 95% confidence interval (CI) for the SAQ scores between the two groups were calculated using Student’s t-test. Superiority was established if the lower bound of the 95% CI was greater than the predefined margin of 1. Categorical variables were expressed as frequencies and percentages (%). To minimize confounding bias in the observational study to the greatest extent possible, a 1:1 propensity score matching (PSM) was employed. Using the nearest-neighbor matching method without replacement and a caliper width of 0.2 of the standard deviation of the logit of the propensity score, propensity scores were calculated based on patients’ exact variables: age, gender, BMI, smoking history, hypertension, diabetes, hyperlipidemia, stroke history, MI history, PCI history, NYHA class, LVEF, and baseline creatinine. Matching quality was assessed beyond standardized mean differences (SMD) by evaluating the overlapping of propensity score density distributions. After matching, the balance of covariates was confirmed by evaluating the absolute standardized mean difference (Absolute SMD < 0.2). Between-group comparisons of continuous variables were performed using the independent samples *t*-test or Wilcoxon rank-sum test, while categorical variables were compared using Fisher’s exact test. For the stability evaluation of the primary outcome (SAQ PL score), the quartile coefficient of dispersion (QCD) was calculated, and Levene’s test for equality of variances was used to compare the difference in dispersion between the two groups. In subgroup analyses, the Hodges-Lehmann estimator was used to calculate and plot forest plots of the 95% confidence intervals (95% CI) for the median differences of effects between the two groups. All bioinformatics data visualizations were implemented using the ggplot2 and pheatmap packages. A two-sided *p* < 0.05 was considered statistically significant. All data processing and statistical analyses were performed using R software (version 4.4).

To account for minor baseline imbalances that persisted after propensity score matching (e.g., preoperative serum creatinine and NYHA class), an Analysis of Covariance (ANCOVA) was employed to evaluate the differences in postoperative peak biomarker levels. Because the biomarker data (creatinine and BNP) were non-normally distributed, they were natural log-transformed prior to ANCOVA modeling to satisfy the assumption of normally distributed residuals. The baseline value was included as a continuous covariate, and the treatment group as a fixed factor.

## 3 Results

### 3.1 Study population enrollment and baseline characteristics

**Figure 1:**
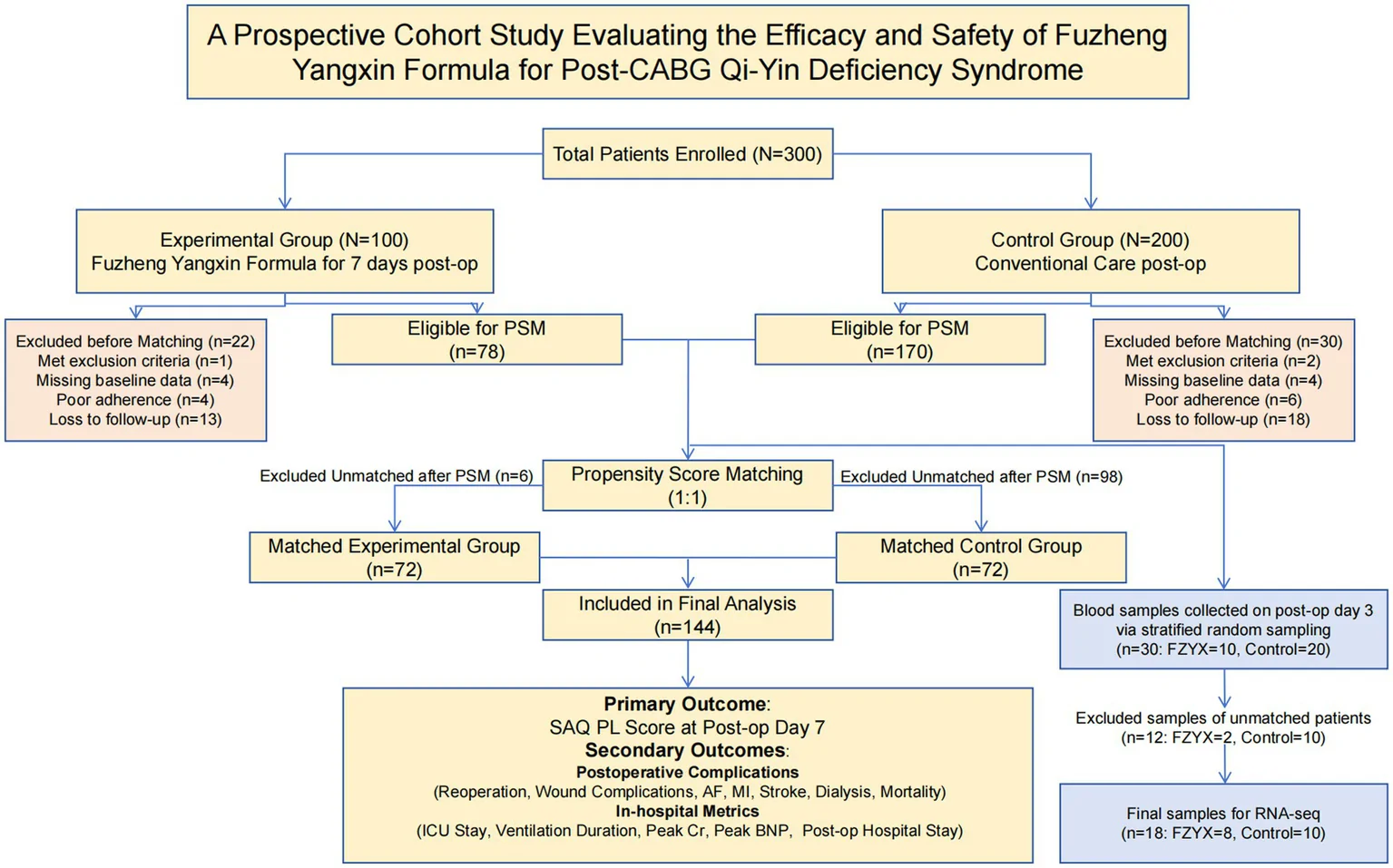
Flowchart of study population enrollment and analysis. This flowchart illustrates the complete process of screening, group allocation, propensity score matching (PSM), and final inclusion of patients post-coronary artery bypass grafting (CABG) diagnosed with Qi-Yin deficiency syndrome. Specific exclusions, loss to follow-up, and the sampling branch detailing how 30 collected blood samples were matched down to 18 final RNA-seq samples are explicitly detailed in the flow branches. FZYX, Fuzheng Yangxin Formula; SAQ PL, Seattle Angina Questionnaire Physical Limitation; AF, Atrial fibrillation; MI, Myocardial infarction; ICU, Intensive care unit; Cr, Creatinine; BNP, B-type natriuretic peptide.

This prospective cohort study initially evaluated 300 patients who underwent CABG and were postoperatively diagnosed with Qi-Yin deficiency syndrome. According to the treatment protocol, 100 patients were allocated to the experimental group (FZYX group) and received consecutive 7-day FZYX granule interventions on top of conventional therapy; the remaining 200 patients were allocated to the control group (Control group) and received only postoperative conventional therapy (Figure 1).

**Figure 2:**
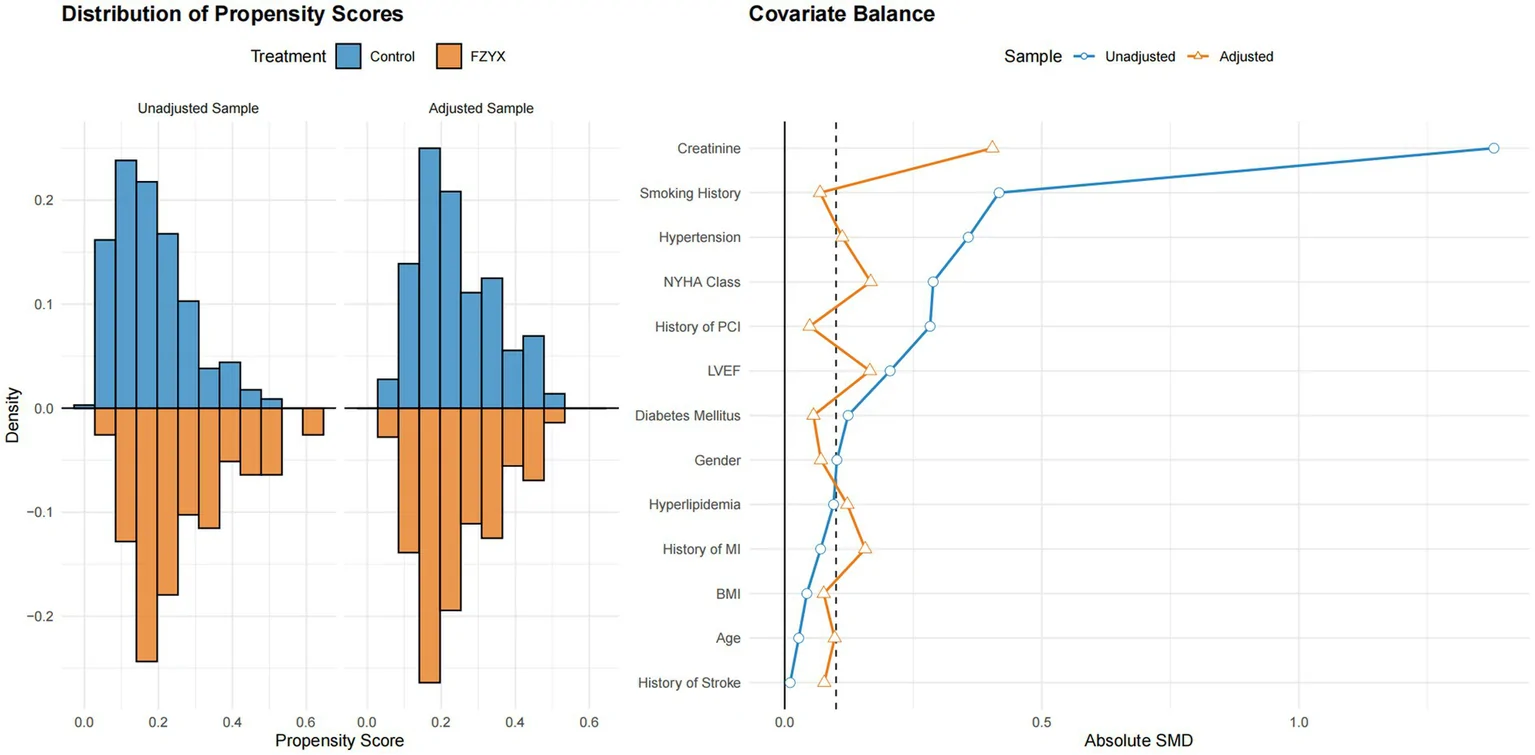
Distribution of propensity scores and covariate balance testing before and after matching. The left panel (Distribution of Propensity Scores) displays the probability density distribution of propensity scores for the control group (Control, blue) and the experimental group (FZYX, orange) before matching (Unadjusted Sample) and after matching (Adjusted Sample). The right panel (Covariate Balance) is a Love plot showing the absolute standardized mean differences (Absolute *SMD*) for each baseline covariate before (blue solid line) and after (orange dashed line) matching. The vertical dashed line represents an absolute *SMD* threshold of 0.2. While the absolute *SMDs* of most covariates were substantially reduced to below 0.2 after matching, minor imbalances persisted for baseline creatinine and NYHA class. To ensure a robust evaluation of postoperative renal function, baseline creatinine was specifically adjusted for in the subsequent downstream ANCOVA model.

Given the observational nature of this cohort study, a 1:1 propensity score matching (PSM) method was employed to adjust for baseline covariates between the two groups to minimize potential confounding biases caused by non-randomized allocation. As shown in Figure 2, there was a noticeable difference in the propensity score distribution between the two groups before matching, and the absolute standardized mean differences (Absolute SMD) of some key covariates (such as serum creatinine levels and smoking history) were relatively large, suggesting baseline imbalance. After PSM adjustment, the probability density distributions of the propensity scores of the two groups highly overlapped, and the absolute SMDs of most covariates significantly decreased to 0.2 or below (Figure 2), demonstrating that matching effectively attenuated the major confounding factors between the groups. Following PSM processing, a total of 144 patients were ultimately included in the primary outcome analysis (*n* = 72 in each of the FZYX and Control groups).

Table 1 details the demographic characteristics and baseline clinical data of the included patients in both groups after PSM. After matching, there were no significant statistical differences between the two groups in mean age (*p* = 0.739), gender composition (the proportion of females was 20.8% in the FZYX group and 18.1% in the control group, *p* = 0.834), and body mass index (BMI, *p* = 0.627). Regarding past medical history and comorbidities, the distributions of smoking history, hypertension, diabetes mellitus, hyperlipidemia, history of stroke, history of myocardial infarction (MI), and history of percutaneous coronary intervention (PCI) were highly consistent between the two groups (all *p* > 0.05).

**Table 1:** Baseline characteristics after propensity score matching.

Characteristic	Control	FZYX	*p* value	SMD
Number	72	72		
Age (years)	67.54 [57.40, 72.17]	67.54 [59.48, 71.69]	0.739	0.094
Female	13 (18.1)	15 (20.8)	0.834	0.070
BMI (kg/m^2^)	25.85 (3.64)	25.55 (3.77)	0.627	0.081
Smoking	18 (25.0)	16 (22.2)	0.845	0.065
Hypertension	48 (66.7)	44 (61.1)	0.603	0.116
Diabetes	31 (43.1)	33 (45.8)	0.867	0.056
Hyperlipidemia	27 (37.5)	23 (31.9)	0.600	0.117
Stroke	14 (19.4)	12 (16.7)	0.829	0.072
MI	12 (16.7)	17 (23.6)	0.406	0.174
PCI	6 (8.3)	7 (9.7)	1.000	0.048
NYHA Class			0.401	0.285
I	18 (25.0)	16 (22.2)		
II	49 (68.1)	45 (62.5)		
III	4 (5.6)	10 (13.9)		
IV	1 (1.4)	1 (1.4)		
LVEF (%)	68.00 [65.00, 71.00]	66.00 [62.75, 70.00]	0.072	0.161
Creatinine (μmol/L)	83.50 [73.25, 97.00]	80.50 [72.00, 94.00]	0.389	0.290

Values are presented as mean (SD) for normal variables, median [IQR] for non-normal variables, or *n* (%) for categorical variables. *p*-values are calculated using Student’s *t*-test or Wilcoxon rank-sum test for continuous variables, and Fisher’s exact test for categorical variables. SMD: standardized mean difference.

BMI, body mass index; MI, myocardial infarction; PCI, percutaneous coronary intervention; NYHA, New York Heart Association; LVEF, left ventricular ejection fraction.

Furthermore, key preoperative baseline indicators reflecting cardiac and target organ functions, including the New York Heart Association (NYHA) functional classification (*p* = 0.401), left ventricular ejection fraction (LVEF, *p* = 0.072), and preoperative serum creatinine levels (*p* = 0.389), also achieved good statistical balance between the groups. These results demonstrate that major confounding factors were effectively attenuated. Although minor imbalances persisted for baseline creatinine and NYHA class (absolute SMD > 0.2), baseline creatinine was specifically included as a covariate in the downstream ANCOVA model to ensure a robust outcome evaluation for peak postoperative creatinine.

### 3.2 Postoperative clinical outcomes and safety evaluation

**Figure 3:**
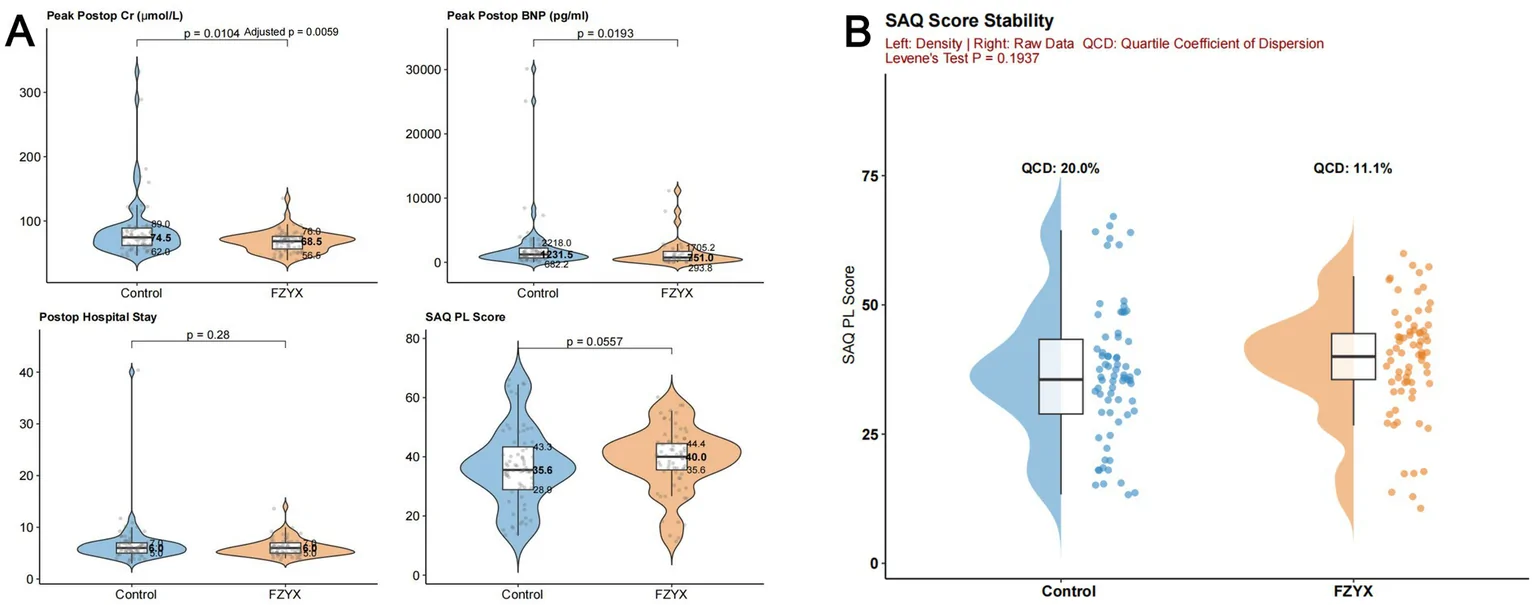
Comparison of postoperative key biomarkers and quality of life scores between the two patient groups. **(A)** Violin plots combined with box plots demonstrate the distributional differences in peak Postop serum creatinine (Peak Postop Cr), peak B-type natriuretic peptide (Peak Postop BNP), postoperative hospital stay, and SAQ physical limitation score (SAQ PL Score) after 7 days of intervention between the two groups. The box plots show the median and interquartile range. The FZYX group exhibited statistically significant differences in lowering the peak values of postoperative Cr (*p* = 0.010; adjusted *p* = 0.0059) and BNP (*p* < 0.05). The adjusted *p*-value for peak Cr was derived from an Analysis of Covariance (ANCOVA) using log-transformed data to adjust for baseline preoperative creatinine disparities. **(B)** SAQ PL score stability analysis. The left side shows the data density distribution, and the right side presents the raw data scatter plot. The quartile coefficient of dispersion (QCD) for the FZYX group was 11.1%, which is noticeably lower than the 20.0% in the control group (Levene’s test *p* = 0.1937), suggesting that the recovery of postoperative physical limitation in the experimental group was more stable and concentrated.

The primary outcome measure of this study was the Seattle Angina Questionnaire Physical Limitation (SAQ PL) score after 7 days of intervention. In the predefined superiority analysis, the mean SAQ PL score was 39.35 in the FZYX group and 36.81 in the control group. The mean difference between the two groups was 2.54 (95% CI: −1.45 to 6.54, *p* = 0.210). Because the lower bound of the 95% CI (−1.45) was below the predefined superiority margin of 1, the FZYX intervention did not meet the strict statistical criteria for superiority. However, based on the non-parametric evaluation, the median SAQ PL score in the FZYX group (40.00 [IQR: 35.56, 44.44]) exhibited a clear trend toward improvement compared to the control group (35.56 [IQR: 28.89, 43.33], *p* = 0.055). As a prospective exploratory study, this potential trend in quality-of-life improvement provides crucial baseline data for sample size estimation in future large-scale randomized controlled trials (RCTs). Furthermore, an analysis of the dispersion of the SAQ PL scores (Figure 3B) revealed that the quartile coefficient of dispersion (QCD) in the FZYX group was 11.1%, noticeably lower than the 20.0% observed in the control group. Although Levene’s test for equality of variances showed no significant difference (*p* = 0.1937), the lower QCD reflects that patients in the experimental group demonstrated more concentrated and stable recovery of early postoperative physical activity, with smaller inter-individual recovery variances.

Regarding key biomarkers reflecting perioperative target organ stress and functional recovery, the FZYX group demonstrated significant advantages (Figure 3A, Table 2). Compared to the control group, peak postoperative serum creatinine levels were significantly lower in the FZYX group (68.50 [56.50, 76.00] μmol/L vs. 74.50 [62.00, 89.00] μmol/L, *p* = 0.010; adjusted *p* = 0.0059), suggesting that FZYX may possess a renoprotective effect in the early post-CABG period. Concurrently, peak postoperative B-type natriuretic peptide (BNP) levels also showed a significant decrease in the FZYX group (751.00 [293.75, 1705.25] pg/ml vs. 1231.50 [682.25, 2218.00] pg/ml, *p* = 0.019). This result indicates that patients in the experimental group had reduced ventricular wall tension and cardiac load post-surgery, leading to better functional recovery.

**Table 2:** Postoperative outcomes in the propensity score-matched cohort.

Outcome	Control	FZYX	P Value
Number	72	72	
SAQ PL Score	35.56 [28.89, 43.33]	40.00 [35.56, 44.44]	0.055
ICU Stay (hours)	23.00 [19.00, 39.50]	24.00 [20.00, 40.00]	0.563
Mechanical Ventilation (hours)	7.00 [5.00, 11.00]	8.00 [4.00, 12.75]	0.810
Peak Postop Cr (μmol/L)	74.50 [62.00, 89.00]	68.50 [56.50, 76.00]	0.010
Peak Postop BNP (pg/ml)	1231.50 [682.25, 2218.00]	751.00 [293.75, 1705.25]	0.019
Postop Hospital Stay (days)	6.00 [5.00, 7.00]	6.00 [5.00, 7.00]	0.279
Reoperation (Thoracotomy)	1 (1.4)	0 (0.0)	1.000
Wound Complications	0 (0.0)	0 (0.0)	NA
Atrial Fibrillation	0 (0.0)	0 (0.0)	NA
Myocardial Infarction	6 (8.3)	11 (15.3)	0.302
Stroke	1 (1.4)	0 (0.0)	1.000
Renal Failure Requiring Dialysis	0 (0.0)	0 (0.0)	NA
Mortality	0 (0.0)	0 (0.0)	NA

Values are presented as median [IQR] for continuous variables or *n* (%) for categorical variables. *p*-values are calculated using Wilcoxon rank-sum test or Fisher’s exact test.

SAQ PL, Seattle Angina Questionnaire Physical Limitation; ICU, intensive care unit; Cr, creatinine; BNP, brain natriuretic peptide; NA, not applicable.

In terms of routine postoperative recovery metrics, no significant differences were observed between the two groups in ICU stay (FZYX group 24.00 h vs. Control group 23.00 h, *p* = 0.563), mechanical ventilation time (8.00 h vs. 7.00 h, *p* = 0.810), and total postoperative hospital stay (both medians 6.00 days, *p* = 0.279) (Table 2). For safety evaluation, neither group experienced postoperative poor wound healing, new-onset atrial fibrillation, renal failure requiring dialysis, or death. The incidence of postoperative myocardial infarction (MI) was 11 cases (15.3%) in the FZYX group and 6 cases (8.3%) in the control group, with no statistically significant difference (*p* = 0.302). The control group had 1 case of reoperation (thoracotomy) and 1 case of stroke, whereas the FZYX group had 0 cases for both (*p* = 1.000). Overall, the addition of FZYX to conventional Western medical therapy did not increase the risk of perioperative complications, demonstrating a favorable clinical safety profile.

### 3.3 Subgroup analysis and exploration of potential benefiting populations

**Figure 4:**
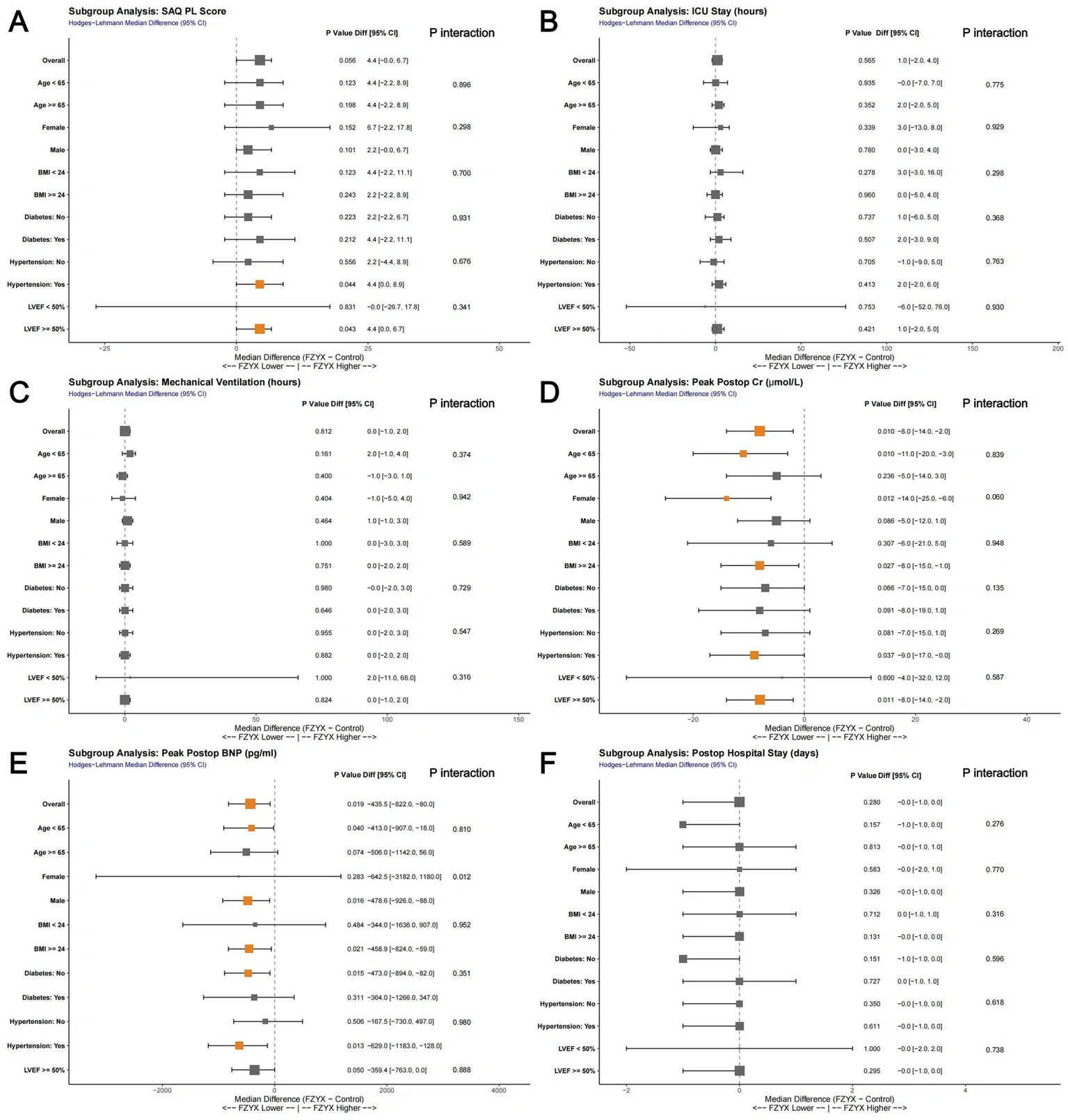
Forest plots of subgroup analyses for major postoperative clinical outcomes and biomarkers. The Hodges-Lehmann median difference was used to evaluate the effect size difference between the two groups. The squares in the figure represent the median difference (FZYX group - Control group), and the horizontal lines represent the 95% confidence intervals (CI). The “*P* for interaction” is presented on the right side of each sub-plot to test the consistency of the treatment effect. **(A–F)** Subgroup analyses of SAQ PL score, ICU stay, mechanical ventilation time, peak postoperative Cr, peak postoperative BNP, and total postoperative hospital stay. Overall, the treatment effects were highly consistent across most prespecified subgroups (all *P* for interaction > 0.05). A significant treatment-by-sex interaction was only observed for peak postoperative BNP **(E)**.

To evaluate the consistency of the treatment effect across prespecified baseline characteristics, we conducted subgroup analyses for the primary and secondary outcomes, incorporating formal interaction testing to prevent spurious claims of subgroup effects (Figure 4). Overall, the treatment effects of FZYX compared with the Control group were highly consistent across most subgroups, with no significant treatment-by-subgroup interactions detected (all P for interaction > 0.05 for SAQ score, Max Creatinine, ICU time, Ventilation time, and Post-op LOS).

However, a significant treatment-by-sex interaction was observed exclusively for the maximum BNP levels (P for interaction = 0.012). While FZYX showed a significant reduction in Max BNP among male patients (*p* = 0.016), this effect was not statistically significant in female patients (*p* = 0.283), suggesting that sex may modify the treatment effect on postoperative BNP levels.

### 3.4 Postoperative peripheral blood transcriptomic profiles and potential molecular mechanisms

**Figure 5:**
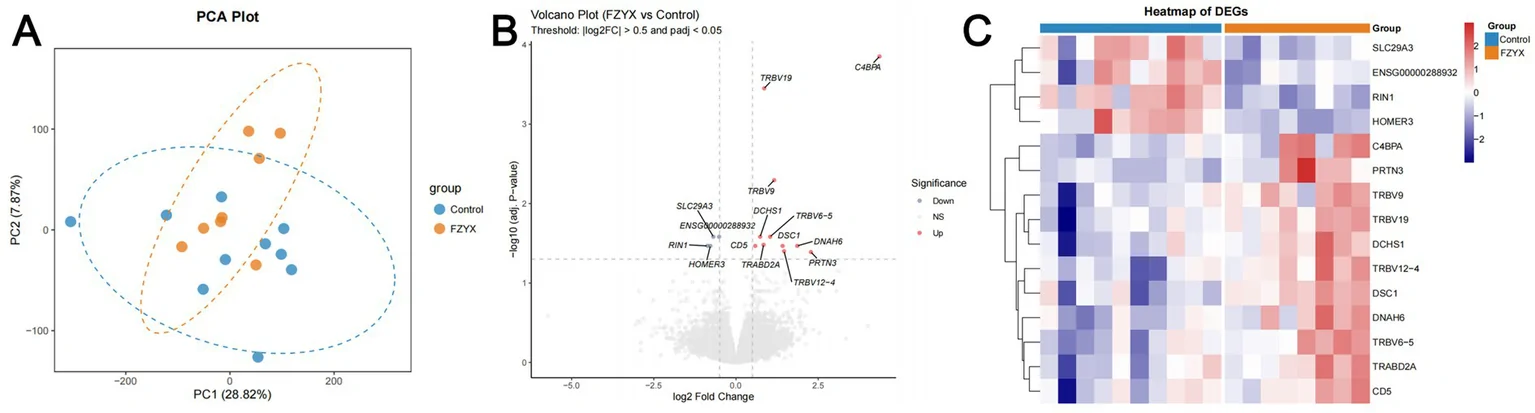
Analysis of postoperative peripheral blood transcriptomic profiles of patients in the two groups. **(A)** Principal component analysis (PCA) plot demonstrating the differences in overall gene expression patterns between the two sample groups. The dashed lines represent 95% confidence ellipses. Although partial overlap exists, the PCA plot demonstrates a distinct separation trend and distributional differences in the overall gene expression patterns between the two groups. **(B)** Volcano plot showing differentially expressed genes (DEGs) between the FZYX and control groups. The screening threshold was set to |*log_2_FC*| > 0.5 and *padj* < 0.05. Red dots represent 11 significantly upregulated genes, and blue dots represent 4 significantly downregulated genes. **(C)** Clustered heatmap visually displaying the relative expression level differences of these 15 significant DEGs in both patient groups, with colors ranging from blue to red indicating low to high expression levels.

To further explore the potential molecular mechanisms by which FZYX promotes enhanced recovery in post-CABG patients with Qi-Yin deficiency syndrome, this study collected peripheral blood samples from patients on postoperative day 3 of the intervention and performed whole-transcriptome RNA sequencing (RNA-seq). Principal component analysis (PCA) results (Figure 5A) revealed a distinct separation trend in the overall gene expression patterns between the two groups. Although the sample distributions of the two groups exhibited partial overlap, the PCA plot indicated a distinct separation trend in the overall gene expression patterns between the two groups.

Through differential expression analysis, we identified a total of 15 statistically significant differentially expressed genes (DEGs) (Figures 5B,C). Among them, 11 genes were significantly upregulated in the FZYX group (C4BPA, PRTN3, DNAH6, TRBV12-4, DSC1, TRBV9, TRBV6-5, TRBV19, TRABD2A, DCHS1, and CD5), while 4 genes were significantly downregulated (ENSG00000288932, SLC29A3, HOMER3, and RIN1). Integrating gene functions with the clinical pathological context, these targets synergistically act on key nodes of cardiovascular postoperative recovery, reflecting a molecular profile characterized by “inhibiting excessive injury, rebuilding immune defense, and initiating tissue repair”:

*Potent anti-inflammation and injury control*: Although minimally invasive cardiac surgery minimizes reliance on cardiopulmonary bypass, local tissue trauma and cardiac manipulation still trigger systemic inflammatory responses and overactivation of the complement system, leading to myocardial ischemia–reperfusion injury. Sequencing results revealed a highly significant upregulation (log_2_FC > 4, *p* < 0.001) of C4BPA, a critical inhibitor of the classical complement pathway (23). This endogenous protective mechanism acts as a “brake,” suppressing complement overactivation and significantly mitigating secondary tissue and endothelial damage.

*Reversing immune paralysis and rebuilding defense*: Following major surgery, patients often encounter an “immune paralysis phase” characterized by impaired T-cell function, making them highly susceptible to concurrent infections. The collective upregulation of multiple T-cell receptor variable region genes (such as TRBV19, TRBV9, TRBV12-4, etc.) and the pan-T cell marker CD5 in the FZYX group signals a robust resurgence of the adaptive immune system (24). Furthermore, the significant upregulation of PRTN3, which participates in neutrophil defense, further enhances the body’s capacity to clear pathogens and necrotic tissue debris, effectively helping patients overcome the postoperative immunosuppressive phase (25).

*Accelerating tissue healing and structural remodeling*: Wound healing is highly dependent on the reconstruction of intercellular tight junctions. The significant upregulation of DSC1 and DCHS1, members of the cadherin superfamily, structurally fortifies intercellular adhesion complexes at the molecular level, which is beneficial for maintaining tissue integrity and accelerating the closure of incisions and vascular anastomoses (26). Concurrently, the upregulation of DNAH6, closely related to ciliary motility, may aid in improving the ciliary clearance function of the postoperative respiratory epithelium, promoting sputum expectoration, and thereby reducing the risk of postoperative pulmonary complications (27).

*Optimizing cellular signaling and promoting survival*: Negative regulatory factors of key survival signaling pathways (such as RIN1, which inhibits the Ras/MAPK pathway) and HOMER3, involved in the regulation of intracellular calcium homeostasis, were significantly downregulated (28, 29). The downregulation of these negative targets essentially removes the body’s suppression of cell survival signals and helps prevent intracellular calcium overload induced by ischemia–reperfusion, creating a highly favorable microenvironment for the survival and metabolic fine-tuning of injured cardiomyocytes.

In summary, the therapeutic mechanism of FZYX is not merely single-target anti-inflammation, but rather a multidimensional remodeling of the postoperative systemic transcriptional network achieved through precise regulation of complement immunity, restoration of T-cell receptor diversity, fortification of structural tissue connections, and optimization of cell survival signals. This provides solid molecular biological evidence for its clinical efficacy in reducing target organ stress and accelerating physical functional recovery.

### 3.5 Multi-dimensional biological mechanisms of recovery revealed by gene set enrichment analysis (GSEA)

**Figure 6:**
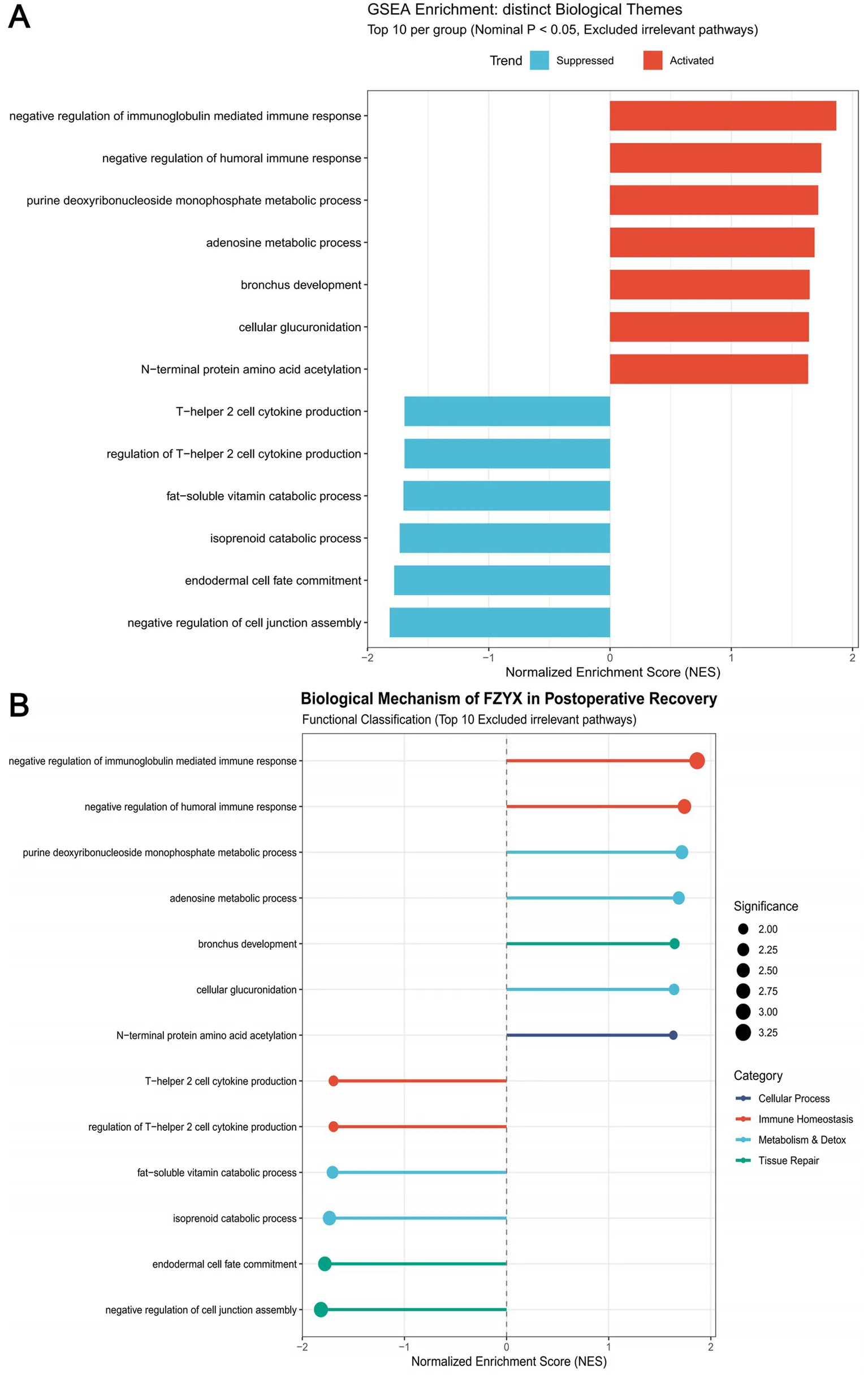
Gene set enrichment analysis (GSEA) and functional classification after FZYX intervention. **(A)** The GSEA bar chart displays the top 10 significantly activated (red) and significantly suppressed (blue) biological pathways based on normalized enrichment scores (NES) in the FZYX group (nominal *p* < 0.05). Pathways lacking biological plausibility in the context of CABG were manually excluded to strictly focus on surgical prognosis. **(B)** Lollipop chart of pathway classifications based on core biological functions. The significant pathways were categorized into four major modules: Immune homeostasis, Tissue repair, Metabolism & detox, and Cellular process. The horizontal axis represents the real NES values, the size of the dots represents statistical significance (−log_10_ *p* value), and line colors represent different functional categories.

To elucidate the molecular mechanisms by which FZYX promotes enhanced recovery in post-CABG patients with Qi-Yin deficiency syndrome from a global transcriptional network perspective, this study utilized Gene Set Enrichment Analysis (GSEA) to conduct unbiased pathway enrichment evaluation of all sequenced transcripts. The results identified 20 highly representative significantly altered pathways (Figure 6A, nominal *p* < 0.05). The functional classification network indicates (Figure 6B) that FZYX does not act upon a single target; rather, functioning as a multidimensional “systemic modulator,” it precisely regulates four core functional modules during postoperative recovery.

Regarding immune homeostasis remodeling, FZYX effectively attenuated postoperative immune over-stress. Results showed that FZYX significantly activated the “negative regulation of humoral immune response” and “negative regulation of immunoglobulin mediated immune response” pathways, while significantly suppressing the production of Th2 pro-inflammatory cytokines and their regulatory pathways. This implies that when confronting the extremely active systemic inflammation following major cardiac surgery, FZYX did not adopt a simplistic “immune enhancement” strategy. Instead, it precisely engaged the immune system’s “braking mechanism,” effectively preventing delayed tissue damage caused by postoperative immune system overactivation.

Concerning barrier repair and tissue remodeling, FZYX accelerated wound healing via specific molecular mechanisms. GSEA results revealed that FZYX significantly suppressed the “negative regulation of cell junction assembly” pathway. This molecular behavior of lifting negative constraints essentially cleared obstacles to the assembly of tight junctions in the early postoperative period, thereby directly driving the reconstruction of physical barriers (e.g., vascular endothelium, tissue incisions). Additionally, morphogenesis pathways related to bone and connective tissues were significantly activated, forecasting that the damaged tissues were undergoing high-quality structural remodeling.

In terms of metabolic optimization and detoxification, FZYX fostered a favorable metabolic microenvironment. Given that the postoperative microenvironment often accumulates massive metabolic waste and drug residues, FZYX significantly activated a vital detoxification pathway—cellular glucuronidation—and upregulated the adenosine metabolic process, which can improve tissue microcirculation and combat hypoxia. Even more critically, FZYX significantly suppressed the catabolic process pathways for fat-soluble vitamins and isoprenoids. This potent inhibition of catabolism implies that FZYX can help extremely debilitated postoperative patients avoid the excessive consumption of essential repair materials, allowing them to smoothly transition into a “nutrient accumulation” state more conducive to tissue regeneration.

At the level of cellular homeostasis, FZYX also exhibited potential systemic maintenance effects by activating the N-terminal protein amino acid acetylation pathway, which helps maintain the stability of recovery-related proteins. Collectively, GSEA reveals that FZYX provides a clear biological mechanistic explanation for enhanced recovery post-CABG through a comprehensive molecular rationale of “quelling inflammation, removing repair obstacles, and accelerating detoxification and nutrient accumulation.”

### 3.6 Characteristics of postoperative peripheral blood immune cell infiltration and their correlation with key genes

**Figure 7:**
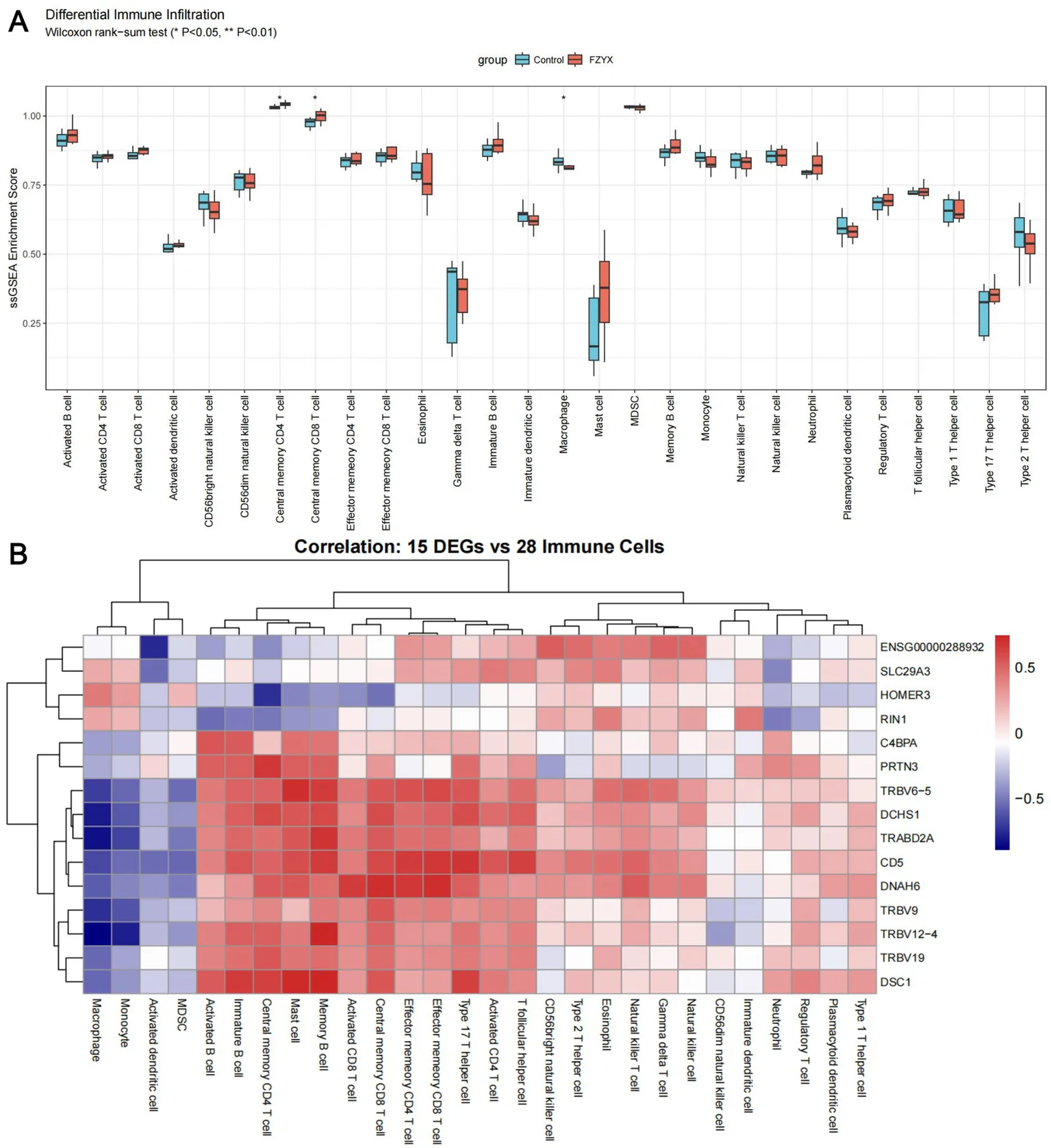
Differences in peripheral blood immune cell infiltration abundance and their correlation analysis with key DEGs between the two patient groups. **(A)** Box plots of the infiltration abundance of 28 immune cell types based on the ssGSEA algorithm. Single-sample gene set enrichment analysis (ssGSEA) was utilized to quantify the relative enrichment scores of 28 immune cell subpopulations in the peripheral blood of each patient, with between-group comparisons performed using the Wilcoxon rank-sum test (*p < 0.05, **p < 0.01). Compared to the control group, the estimated abundance of Central memory CD4 T cells and Central memory CD8 T cells in the FZYX group was significantly elevated, while the estimated abundance of the monocyte/macrophage lineage was significantly decreased. **(B)** Clustered heatmap of Spearman correlations between 15 key DEGs and the abundance of 28 immune cells. Red indicates a positive correlation, blue indicates a negative correlation, and the color intensity reflects the magnitude of the correlation coefficient. Clustering results demonstrate that downregulated genes (e.g., SLC29A3, HOMER3, RIN1) highly positively correlated with innate immune cells such as macrophages, whereas the upregulated gene cluster highly positively correlated with adaptive immune cells such as central memory T cells.

Building upon the “remodeling of immune homeostasis” signals emerged from the aforementioned DEG and GSEA analyses, this study further utilized the ssGSEA algorithm to precisely quantify and compare the infiltration abundance of 28 immune cell subpopulations in the patients’ peripheral blood. As shown in Figure 7A, FZYX intervention significantly altered the composition of the patients’ postoperative immune microenvironment. In terms of the adaptive immune defense line, the infiltration abundances of central memory CD4 T cells and central memory CD8 T cells in the peripheral blood of the FZYX group exhibited statistically significant increases compared to the control group (both *p* < 0.05). This targeted expansion of memory T cells signifies that the body rapidly reconstructed specific immune surveillance and long-term defense capabilities against potential pathogens post-surgery. Conversely, regarding the innate inflammatory response, the estimated abundance of the monocyte/macrophage lineage in the peripheral blood of the FZYX group was significantly downregulated (*p* < 0.05). Since macrophages are the core driving factors that trigger systemic inflammatory response syndrome (SIRS) and tissue injury in the early postoperative period, their significant proportional decrease further corroborates that FZYX exerted precise “anti-inflammation and injury control” effects in the early postoperative phase.

To investigate the intrinsic connections between core molecular expressions and systemic immune phenotypic alterations, this study conducted a Spearman correlation analysis between the 15 identified key DEGs and the abundance of 28 immune cells (Figure 7B). The clustered heatmap revealed two distinct gene-immune cell association patterns. On one hand, the four genes significantly downregulated in the FZYX group (including SLC29A3, HOMER3, RIN1, and ENSG00000288932) displayed strong positive correlations with innate pro-inflammatory or immunosuppressive cell communities, represented by macrophages, monocytes, and myeloid-derived suppressor cells (MDSCs). This implies that FZYX’s suppression of these target genes phenotypically corresponds to the effective attenuation of pro-inflammatory macrophage activity. On the other hand, the 11 genes significantly upregulated in the FZYX group (such as the TRBV family, CD5, C4BPA, etc.) exhibited highly positive correlations with adaptive immune cell communities like central memory CD4 T cells and central memory CD8 T cells. This strong correlation indicates that the upregulation of key genes targeting T-cell receptor signaling and immunomodulation is the core molecular driver promoting the remodeling of the postoperative specific immune defense line.

Synthesizing the above immune phenotyping and correlation analysis results, it is evident that FZYX, through precise regulation of the expression of specific gene clusters, effectively shifted the immune status of post-CABG patients in a beneficial polarized transition from a “macrophage-dominated innate destructive inflammation” toward a “central memory T cell-dominated adaptive protective immunity.” This provides robust dual cytological and molecular evidence for the rapid restoration of bodily homeostasis post-surgery.

## 4 Discussion

This study is the first prospective cohort study aimed at evaluating the application value of an integrative medicine strategy—the Fuzheng Yangxin Formula (FZYX)—in the enhanced recovery after surgery (ERAS) of post-coronary artery bypass grafting (CABG) patients with Qi-Yin deficiency syndrome. Through rigorous propensity score matching (PSM) combined with peripheral blood whole-transcriptome sequencing analysis, our study confirms that, without increasing perioperative complications, FZYX was associated with significantly reduced key biomarkers reflecting target organ stress. Furthermore, it showed a trend toward improving the recovery trajectory of early postoperative physical limitation, effectively smoothing out inter-individual recovery variances. Multi-dimensional omics analysis further generated the hypothesis that these clinical benefits may be associated with FZYX’s remodeling of the systemic “immune-inflammatory” network, particularly its synergistic targeted effects in suppressing innate macrophage inflammation, restoring adaptive memory T cells, and accelerating the physical repair of cell tight junctions.

In recent years, with continuous advancements in surgical techniques and perioperative management, the concept of ERAS after CABG has become an important development direction in cardiovascular surgery. However, regardless of how surgical approaches are optimized, myocardial ischemia–reperfusion injury, tissue trauma, and the inevitable systemic micro-inflammatory stress induced by cardiac surface manipulations persist. These pathological factors often lead to transient target organ damage and physical limitation in patients during the early postoperative period. Our results show that, on top of conventional standard therapy, the peak postoperative serum creatinine and B-type natriuretic peptide (BNP) levels in the FZYX group remained significantly lower than those in the conventional therapy alone control group. This finding suggests that FZYX provides a protective effect on renal function and potential protective effects on other target organs beyond conventional surgical interventions, effectively buffering the physiological and ischemic stresses that are hard to entirely avoid during surgery. This echoes the findings of multiple previous studies highlighting the efficacy of TCM formulas in improving microcirculation and organ protection, underscoring its immense potential in optimizing the perioperative internal milieu in cardiac surgery.

Regarding the primary quality-of-life outcome, although it did not meet the criteria for superiority testing, the non-parametric test of the SAQ Physical Limitation (PL) score after 7 days of FZYX intervention indicated a trend of improvement (*p* = 0.055), providing a basis for sample size calculation for subsequent randomized controlled trials (RCTs). Dispersion analysis revealed that the quartile coefficient of dispersion (QCD) for the scores in the FZYX group was only 11.1%, far lower than the 20.0% observed in the control group. In the core philosophy of postoperative ERAS, reducing the variance in the recovery trajectory is just as important as improving absolute scores. FZYX significantly narrowed inter-individual recovery differences, making the recovery of physical function in CABG patients more “homogenized” and stable, thereby effectively preventing some patients from experiencing a delayed recovery phase due to extreme frailty. Furthermore, subgroup analyses suggested that the clinical benefits of FZYX were more pronounced in patients with concomitant hypertension, a heavier body build (BMI ≥ 24 kg/m^2^), and a relatively younger age (<65 years). These populations typically present with higher baseline metabolic disturbances and endothelial inflammatory states, suggesting that FZYX exerts superior buffering capacity in specific patient phenotypes dealing with high baseline inflammatory loads.

Furthermore, our comprehensive subgroup analyses confirmed that the clinical benefits of FZYX are broadly applicable across diverse patient demographics. While we noted a significant interaction between sex and treatment regarding maximum BNP levels (P for interaction = 0.012)—generating a compelling hypothesis regarding sex-specific pathophysiological responses—this must be interpreted with caution. Subgroup analyses are inherently susceptible to false-positive findings due to multiple comparisons. Therefore, this exploratory finding warrants further validation in adequately powered, sex-stratified prospective studies.

The molecular mechanisms underlying these clinical observations were further elucidated at the molecular biology level through peripheral blood whole-transcriptome sequencing and ssGSEA immune phenotyping. CABG surgical trauma (and potentially accompanied cardiopulmonary bypass) typically triggers innate immune over-stress dominated by macrophages and subsequent immune exhaustion. Our results showed that FZYX not only significantly reduced the infiltration abundance of macrophages in peripheral blood, but this decrease was also highly correlated with the significant suppression of pro-inflammatory regulatory genes such as SLC29A3. Even more critically, C4BPA, a potent inhibitor of the classical complement pathway, exhibited an extremely significant upregulation (log_2_FC > 4). This mechanism acts precisely as a “brake” on the excessive innate inflammatory response post-surgery, providing a mechanistic basis for the protective effect on renal function and the potential protective effects on the heart in the FZYX group. Concurrently, it is consistent with the modern pharmacological mechanisms suggesting that core active components in the formula, such as Ginsenoside Rg1 and Salidroside, may regulate macrophage polarization.

While potently inhibiting excessive inflammation, FZYX actively restarts the body’s protective and repair mechanisms. Immune omics data demonstrated that the proportions of central memory CD4+ and CD8+ T cells in the FZYX group increased significantly, and showed a strong positive correlation with the upregulation of TRBV family genes and the pan-T cell marker CD5. This indicates that the medication helped patients successfully reverse the potential postoperative “immune paralysis” state, rebuilding a specific immune defense line against potential nosocomial infections. Furthermore, the “double negative” mechanism (lifting the negative inhibition on cell junction assembly) revealed in the GSEA analysis, combined with the significant upregulation of DSC1 and DCHS1 (core adhesion molecules constituting desmosomes), provided preliminary molecular support for the potential of FZYX in accelerating wound healing and fortifying the integrity of tissue incisions and vascular anastomoses. This is highly consistent with modern pharmacological understandings that components like Astragalus in the TCM formula promote tissue repair, and also provides potential molecular insights into the traditional classic TCM theory of “replenishing Qi and activating blood, expelling toxins and generating flesh.”

The integration of blood-based transcriptomics with TCM clinical outcomes provides a modern interpretable framework for traditional formulas. Similar multi-omics and network pharmacology strategies have been successfully applied to decode the blood molecular targets of other herbal medicines (30) and biomarker-driven approaches in oncology (31). Furthermore, our finding regarding the downregulation of macrophage/monocyte-associated inflammatory targets aligns with recent advances in immune signaling, where modulating macrophage polarization (e.g., via Galectin-9 pathways) is increasingly recognized as a crucial therapeutic strategy (32, 33).

### 4.1 Limitations

Several limitations of this study must be acknowledged. First, due to the single-center, non-randomized observational design, patients were allocated based on their willingness to receive TCM. Despite rigorous PSM adjusting for 13 baseline covariates, residual confounding (e.g., patient expectations, health behaviors, unmeasured perioperative variables like exact fluid infusion volumes, operation time, and surgeon experience) cannot be entirely eliminated, and expectation effects may have influenced patient-reported outcomes (SAQ scores). Second, the primary clinical endpoint (SAQ-PL at 7 days) did not achieve strict statistical significance for superiority, and causal relationships should not be definitively inferred from observational data. Third, the RNA-seq analysis was performed on a small subset (*n* = 18) and lacked pretreatment baseline samples; therefore, the transcriptomic findings and computational ssGSEA immune estimates should be interpreted strictly as exploratory and hypothesis-generating. Direct immunophenotyping via flow cytometry and external validation in independent cohorts are required to confirm these molecular mechanisms. Fourth, to avoid the severe inflammatory interference of cardiopulmonary bypass (CPB), the cohort was strictly limited to off-pump minimally invasive CABG patients, limiting the generalizability of the findings to conventional on-pump procedures. Finally, given that FZYX contains Aconitum, the 7-day short-term observation lacks detailed quantitative data on continuous electrocardiographic monitoring (e.g., QTc interval, ventricular arrhythmias) and liver function, which necessitates longer-term, comprehensive safety profiling in future multicenter RCTs.

## 5 Conclusion

FZYX appears to be a safe and promising adjunctive therapy that may provide post-CABG patients with additional clinical benefits beyond conventional therapy. It may not only exert potential renoprotective and broader target organ protective effects, but also promote a more stable and consistent physical recovery during the early postoperative period. Its core mechanism involves mitigating systemic and local inflammatory stress by upregulating C4BPA and downregulating pro-inflammatory macrophage activity, while simultaneously driving adaptive immune reconstruction and tissue structural repair via memory T cell activation and cell junction protein upregulation. This study provides valuable mechanistic insights and preliminary evidence supporting the integration of TCM into modern cardiovascular ERAS systems.

## Data Availability

The raw transcriptome sequence data reported in this article have been deposited in the Genome Sequence Archive (GSA-Human) in the National Genomics Data Center, China National Center for Bioinformation / Beijing Institute of Genomics, Chinese Academy of Sciences. The data are associated with the BioProject accession PRJCA057697 and the submission accession HRA017232, and are publicly accessible at https://ngdc.cncb.ac.cn/gsa-human. The clinical datasets generated and analyzed during the current study are available from the corresponding author upon reasonable request.
