# Supplementary material for: Efficacy, safety, and transcriptomic mechanisms of Fuzheng Yangxin formula for enhanced recovery after coronary artery bypass grafting: a propensity score-matched prospective cohort study
Source: Front Med (Lausanne). 2026 Aug 12;13:1902379. doi: 10.3389/fmed.2026.1902379 (PMC13506943; doi:10.3389/fmed.2026.1902379)
Supplement: Supplementary file 1 [file Data_Sheet_1.DOCX]

**Supplementary Figure 1**

**Supplementary Table 1. Chemical composition of FZYX**

| **No.** | **Identification** | **Molecular formula** | **Retention time (min)** | **Adduct Ion** | **Accurate Mass** | **Observed m/z** | **Error (ppm)** |
| --- | --- | --- | --- | --- | --- | --- | --- |
| 1 | L-Canavanine | C₅H₁₂N₄O₃ | 0.76 | [M+H]+ | 176.09094 | 177.0988 | 3.2 |
| 2 | L-Arginine | C₆H₁₄N₄O | 0.77 | [M+H]+ | 174.11168 | 175.1193 | 1.7 |
| 3 | Argininyl-fructosyl-glucose deglycosylation | C₁₂H₂₄N₄O₇ | 0.77 | [M+H]+ | 336.1645 | 337.1714 | -1.2 |
| 4 | 2-Acetylacteoside | C₃₁H₃₈O₁₆ | 0.79 | [M+Na]+ | 666.21599 | 689.2082 | 4.3 |
| 5 | Gentianose | C₁₈H₃₂O₁₆ | 0.79 | [M+Na]+ | 504.16903 | 527.1567 | -3 |
| 6 | beta-Lactose | C₁₂H₂₂O₁₁ | 0.82 | [M+Cl]- | 342.11621 | 377.0859 | 0.8 |
| 7 | Proline | C₅H₉NO₂ | 0.84 | [M+H]+ | 115.06333 | 116.0709 | 2.2 |
| 8 | trans-Aconitic acid | C₆H₆O₆ | 0.89 | [M-H]- | 174.01644 | 173.0095 | 1.9 |
| 9 | Citric acid | C₆H₈O₇ | 0.9 | [M-H]- | 192.027 | 191.0201 | 2 |
| 10 | Adeninenucleoside | C₁₀H₁₃N₅O₄ | 1.1 | [M+H]+ | 267.09675 | 268.1045 | 1.6 |
| 11 | Isocitric acid | C₆H₈O₇ | 1.1 | [M-H]- | 192.027 | 191.02 | 1.5 |
| 12 | DL-Pyroglutamic acid | C₅H₇NO₃ | 1.12 | [M-H]- | 129.04259 | 128.0353 | -0.2 |
| 13 | Catalpol | C₁₅H₂₂O₁₀ | 1.13 | [M+FA-H]- | 362.1213 | 407.1193 | -0.4 |
| 14 | Guanosine | C₁₀H₁₃N₅O₅ | 1.15 | [M+H]+ | 283.09167 | 284.1003 | 4.8 |
| 15 | Gallic acid | C₇H₆O₅ | 1.51 | [M-H]- | 170.02152 | 169.0147 | 2.4 |
| 16 | Rhmannioside D | C₂₇H₄₂O₂₀ | 1.63 | [M+FA-H]- | 686.22694 | 731.2247 | -0.7 |
| 17 | Rehmannioside B | C₂₁H₃₂O₁₅ | 1.81 | [M+FA-H]- | 524.17412 | 569.1727 | 0.6 |
| 18 | Phenylalanine | C₉H₁₁NO₂ | 2.14 | [M+H]+ | 165.07898 | 166.0867 | 2.7 |
| 19 | Geniposidicacid | C₁₆H₂₂O₁₀ | 2.52 | [M-H]- | 374.1213 | 373.1144 | 1.1 |
| 20 | Uralene | C₂₁H₂₀O₇ | 2.61 | [M-H]- | 384.1209 | 383.1119 | -4.5 |
| 21 | p-Coumaric acid | C₉H₈O₃ | 2.86 | [M-H]- | 164.04734 | 163.0404 | 1.9 |
| 22 | Salidroside | C₁₄H₂₀O₇ | 3.03 | [M-H]- | 300.1209 | 299.1139 | 1.1 |
| 23 | Carmichaeline | C₂₂H₃₅NO₄ | 3.19 | [M+H]+ | 377.25661 | 378.2634 | -1.2 |
| 24 | Isotalatizidine | C₂₃H₃₇NO₅ | 3.21 | [M+H]+ | 407.26717 | 408.2739 | -1.2 |
| 25 | Chlorogenic acid | C₁₆H₁₈O₉ | 3.28 | [M-H]- | 354.09508 | 353.0882 | 1.1 |
| 26 | Songorine | C₂₂H₃₁NO₃ | 3.37 | [M+H]+ | 357.23039 | 358.2381 | 1.2 |
| 27 | Cis Caffeic acid | C₉H₈O₄ | 3.62 | [M-H]- | 180.04226 | 179.0356 | 3.4 |
| 28 | Purpureaside C | C₃₅H₄₆O₂₀ | 3.63 | [M-H]- | 786.25824 | 785.2504 | -0.7 |
| 29 | Salicylic acid | C₇H₆O₃ | 3.67 | [M+H]+ | 138.03169 | 139.0396 | 4.9 |
| 30 | Neoline | C₂₄H₃₉NO₆ | 3.73 | [M+H]+ | 437.27774 | 438.2846 | -0.8 |
| 31 | Fuzitine | C₂₀H₂₄NO₄ | 3.78 | [M]+ | 342.17053 | 342.1698 | -0.6 |
| 32 | Panasenoside | C₂₇H₃₀O₁₆ | 3.79 | [M-H]- | 610.15338 | 609.1459 | -0.3 |
| 33 | 7-O-methylisomucronulatol | C₁₈H₂₀O₅ | 3.8 | [M+H]+ | 316.13107 | 317.1395 | 3.6 |
| 34 | Talatisamine | C₂₄H₃₉NO₅ | 3.98 | [M+H]+ | 421.28282 | 422.2896 | -1.1 |
| 35 | Jionoside A1 | C₃₆H₄₈O₂₀ | 4.03 | [M-H]- | 800.27389 | 799.2668 | 0.3 |
| 36 | Genistein | C₁₅H₁₀O₅ | 4.1 | [M+H]+ | 270.05282 | 271.0612 | 4 |
| 37 | Ellagic acid | C₁₄H₆O₈ | 4.14 | [M-H]- | 302.00627 | 300.9993 | 1.1 |
| 38 | Calycosin-7-O-beta-D-glucopyranoside | C₂₂H₂₂O₁₀ | 4.2 | [M+H]+ | 446.1213 | 447.1289 | 0.8 |
| 39 | Verbascoside | C₂₉H₃₆O₁₅ | 4.26 | [M-H]- | 624.20542 | 623.1968 | -2.1 |
| 40 | Jionoside D | C₃₀H₃₈O₁₅ | 4.35 | [M-H]- | 638.22107 | 637.2119 | -2.9 |
| 41 | Isoacteoside | C₂₉H₃₆O₁₅ | 4.42 | [M-H]- | 624.20542 | 623.1966 | -2.4 |
| 42 | Rehmaionoside A | C₁₉H₃₄O₈ | 4.43 | [M+FA-H]- | 390.22537 | 435.2228 | -1.7 |
| 43 | Ferulic acid | C₁₀H₁₀O₄ | 4.44 | [M-H]- | 194.05791 | 193.0507 | 0.4 |
| 44 | Naringin | C₂₇H₃₂O₁₄ | 4.6 | [M-H]- | 580.17921 | 579.1723 | 0.6 |
| 45 | Maackiain | C₁₆H₁₂O₅ | 4.64 | [M-H]- | 284.06847 | 283.0614 | 0.8 |
| 46 | Dimethyl azelate | C₁₁H₂₀O₄ | 4.65 | [M+FA-H]- | 216.13616 | 261.1342 | -0.7 |
| 47 | Notoginsenoside R3 | C₄₈H₈₂O₁₉ | 4.7 | [M+FA-H]- | 962.54503 | 1007.5427 | -0.5 |
| 48 | Notoginsenoside R1 | C₄₇H₈₀O₁₈ | 4.78 | [M+FA-H]- | 932.53447 | 977.5324 | -0.3 |
| 49 | Z-6,7-Epoxyligustilide-SG3 | C₂₂H₃₁N₃O₉S | 4.85 | [M+H]+ | 513.1781 | 514.185 | -0.7 |
| 50 | Kaempferol-7-rhamnoside | C₂₁H₂₀O₁₀ | 4.89 | [M-H]- | 432.10565 | 431.0981 | -0.7 |
| 51 | 6-Feruloylajugol | C₂₅H₃₂O₁₂ | 4.9 | [M-H]- | 524.18938 | 523.1822 | 0.2 |
| 52 | Ginsenoside Re | C₄₈H₈₂O₁₈ | 4.92 | [M+FA-H]- | 946.55012 | 991.5476 | -0.7 |
| 53 | Ginsenoside Rg1 | C₄₂H₇₂O₁₄ | 4.95 | [M+FA-H]- | 800.49221 | 845.4896 | -1 |
| 54 | Hyperoside | C₂₁H₂₀O₁₂ | 5 | [M+H]+ | 464.09548 | 465.1029 | 0.3 |
| 55 | Benzoylmesaconine | C₃₁H₄₃NO₁₀ | 5.05 | [M+H]+ | 589.2887 | 590.2955 | -0.8 |
| 56 | Quercetin | C₁₅H₁₀O₇ | 5.1 | [M+H]+ | 302.04265 | 303.0505 | 2 |
| 57 | Martynoside | C₃₁H₄₀O₁₅ | 5.11 | [M-H]- | 652.23672 | 651.2276 | -2.8 |
| 58 | Formononetin 7-O-Glucoside | C₂₂H₂₂O₉ | 5.2 | [M+H]+ | 430.12638 | 431.1335 | -0.4 |
| 59 | Hokbusine A | C₃₂H₄₅NO₁₀ | 5.32 | [M+H]+ | 603.30435 | 604.3109 | -1.1 |
| 60 | Epigallocatechol-3-gallate | C₂₂H₁₈O₁₁ | 5.43 | [M+FA-H]- | 458.08491 | 503.0828 | -0.6 |
| 61 | 9,10-Dimethoxy-pterocarpan-3-O-β-D-glucopyranoside | C₂₃H₂₆O₁₀ | 5.44 | [M+FA-H]- | 462.1526 | 507.1505 | -0.7 |
| 62 | Ginsenoside Re5 | C₄₂H₇₂O₁₅ | 5.44 | [M-H]- | 816.48712 | 815.4785 | -1.7 |
| 63 | Notoginsenoside D | C₆₄H₁₀₈O₃₁ | 5.48 | [M+FA-H]- | 1372.68746 | 1417.6848 | -0.6 |
| 64 | Benzoylhypaconine | C₃₁H₄₃NO₉ | 5.49 | [M+H]+ | 573.29378 | 574.3007 | -0.6 |
| 65 | Luteolin | C₁₅H₁₀O₆ | 5.49 | [M+H]+ | 286.04774 | 287.0558 | 2.6 |
| 66 | Astraisoflavanin | C₂₃H₂₈O₁₀ | 5.54 | [M-H]- | 464.16825 | 463.1608 | -0.5 |
| 67 | Biochanin A | C₁₆H₁₂O₅ | 5.57 | [M+H]+ | 284.06847 | 285.0763 | 1.8 |
| 68 | Ginsenoside Ra3 | C₅₉H₁₀₀O₂₇ | 5.63 | [M+FA-H]- | 1240.6452 | 1285.6447 | 1 |
| 69 | Astragaloside VI | C₄₇H₇₈O₁₉ | 5.67 | [M+H]+ | 946.51373 | 947.5198 | -1.2 |
| 70 | Malonylginsenoside Ra3 | C₆₂H₁₀₂O₃₀ | 5.69 | [M-H]- | 1326.64559 | 1325.6372 | -0.8 |
| 71 | Notoginsenoside R4 | C₅₉H₁₀₀O₂₇ | 5.76 | [M+FA-H]- | 1240.6452 | 1285.6435 | 0.1 |
| 72 | Notoginsenoside I | C₅₄H₉₂O₂₂ | 5.78 | [M+FA-H]- | 1092.60802 | 1137.6061 | -0.1 |
| 73 | 5,6-Didehydroginsenoside Rb1 | C₅₄H₉₀O₂₃ | 5.83 | [M+FA-H]- | 1106.58729 | 1151.5852 | -0.2 |
| 74 | Ginsenoside Ra1 | C₅₈H₉₈O₂₆ | 5.86 | [M+FA-H]- | 1210.63463 | 1255.6328 | 0 |
| 75 | Ginsenoside Rb1 | C₅₄H₉₂O₂₃ | 5.94 | [M+FA-H]- | 1108.60294 | 1153.6023 | 1 |
| 76 | Malonyl-ginsenoside Rb1 | C₅₇H₉₄O₂₆ | 5.98 | [M-H]- | 1194.60333 | 1193.5976 | 1.3 |
| 77 | Ginsenoside Rf | C₄₂H₇₂O₁₄ | 6 | [M+FA-H]- | 800.49221 | 845.4904 | -0.1 |
| 78 | Ginsenoside Ra2 | C₅₈H₉₈O₂₆ | 6.03 | [M+FA-H]- | 1210.63463 | 1255.6326 | -0.2 |
| 79 | (1S,2R,4S)-Borneol beta-D-glucopyranoside | C₁₆H₂₈O₆ | 6.04 | [M+Na]+ | 316.18859 | 339.178 | 0.4 |
| 80 | Ginsenoside Rb2 | C_53_H_90_O_22_ | 6.06 | [M-H]- | 1124.59785 | 1123.5902 | -0.4 |
| 81 | Malonyl-ginsenoside Rb2 | C₅₆H₉₂O₂₅ | 6.11 | [M-H]- | 1164.59277 | 1163.5854 | -0.1 |
| 82 | Kaempferol | C₁₅H₁₀O₆ | 6.12 | [M-H]- | 286.04774 | 285.0405 | 0 |
| 83 | Ginsenoside-Rg4 deglycosylation | C₃₀H₅₀O₃ | 6.15 | [M+H]+ | 458.376 | 459.3828 | -1.1 |
| 84 | Notoginsenoside R2 | C₄₂H₇₂O₁₅ | 6.15 | [M-H]- | 816.48712 | 815.4792 | -0.8 |
| 85 | Ginsenoside Rc | C_53_H_90_O_22_ | 6.19 | [M-H]- | 1124.59785 | 1123.5898 | -0.7 |
| 86 | (Z)-3-Butylidenephthalide | C₁₂H₁₂O₂ | 6.2 | [M+H]+ | 188.08373 | 189.0917 | 3.8 |
| 87 | Ginsenoside Ro | C₄₈H₇₆O₁₉ | 6.21 | [M-H]- | 956.49808 | 955.4898 | -1.1 |
| 88 | Malonyl-ginsenoside  Rc | C₅₆H₉₂O₂₅ | 6.24 | [M-H]- | 1164.59277 | 1163.5861 | 0.5 |
| 89 | Ginsenoside-Rh4 deglycosylation | C₃₀H₅₀O₃ | 6.28 | [M+H]+ | 458.376 | 459.3827 | -1.3 |
| 90 | Notoginsenoside ST-2 | C₄₃H₇₄O₁₅ | 6.29 | [M-H]- | 830.50277 | 829.4948 | -0.8 |
| 91 | 14-O-Anisoylneoline | C₃₂H₄₅NO₈ | 6.31 | [M+H]+ | 571.31452 | 572.321 | -1.3 |
| 92 | Ginsenoside F1 | C₃₆H₆₂O₉ | 6.37 | [M+FA-H]- | 638.43938 | 683.4363 | -1.8 |
| 93 | Hypaconitine | C₃₃H₄₅NO₁₀ | 6.4 | [M+H]+ | 615.30435 | 616.311 | -1 |
| 94 | Chikusetsusaponin IV | C₄₇H₇₄O₁₈ | 6.4 | [M-H]- | 926.48752 | 925.481 | 0.8 |
| 95 | Tianshic acid | C₁₈H₃₄O₅ | 6.46 | [M-H]- | 330.24062 | 329.2337 | 1.1 |
| 96 | Ginsenoside Rh1 | C₃₆H₆₂O₉ | 6.49 | [M+FA-H]- | 638.43938 | 683.4361 | -2.1 |
| 97 | AstragalosideIV | C₄₁H₆₈O₁₄ | 6.51 | [M+FA-H]- | 784.46091 | 829.4595 | 0.5 |
| 98 | Gypenoside VIII | C₄₈H₈₂O₁₈ | 6.53 | [M+FA-H]- | 946.55012 | 991.5475 | -0.9 |
| 99 | Ginsenoside Rh3 | C₃₆H₆₀O₇ | 6.53 | [M+H]+ | 604.4339 | 605.4405 | -1.1 |
| 100 | Ginsenoside Rh3 deglycosylation | C₃₀H₅₀O₂ | 6.53 | [M+H]+ | 442.38108 | 443.3881 | -0.6 |
| 101 | Malonylginsenoside Rd | C₅₁H₈₄O₂₁ | 6.58 | [M-H]- | 1032.55051 | 1031.543 | -0.2 |
| 102 | Astragaloside III | C₄₁H₆₈O₁₄ | 6.61 | [M+FA-H]- | 784.46091 | 829.4582 | -1.1 |
| 103 | Deoxyaconitine | C₃₄H₄₇NO₁₀ | 6.77 | [M+H]+ | 629.32 | 630.3259 | -2.2 |
| 104 | Methylnissolin | C₁₇H₁₆O₅ | 6.93 | [M+H]+ | 300.09977 | 301.1074 | 1.2 |
| 105 | Astragaloside II | C₄₃H₇₀O₁₅ | 6.95 | [M+H]+ | 826.47147 | 827.4774 | -1.7 |
| 106 | Soyasaponin I | C₄₈H₇₈O₁₈ | 7.05 | [M+FA-H]- | 942.51882 | 987.5151 | -1.9 |
| 107 | Ditertbutyl phthalate | C₁₆H₂₂O₄ | 7.44 | [M+H]+ | 278.15181 | 279.1598 | 2.4 |
| 108 | Ginsenoside Rg4 | C₄₂H₇₀O₁₂ | 7.7 | [M+FA-H]- | 766.48673 | 811.4852 | 0.3 |
| 109 | Ophiopogonanone E | C₁₉H₂₀O₇ | 7.76 | [M+H]+ | 360.1209 | 361.1285 | 0.8 |
| 110 | Isoastragaloside I | C₄₅H₇₂O₁₆ | 7.77 | [M+H]+ | 868.48204 | 869.4884 | -1.1 |
| 111 | 28-Deglucosyl chikusetsusaponin V | C₄₂H₆₆O₁₄ | 7.79 | [M-H]- | 794.44526 | 793.4366 | -1.7 |
| 112 | Ophiopojaponin A | C₄₆H₇₂O₁₈ | 8 | [M+FA-H]- | 912.47187 | 957.4682 | -2 |
| 113 | Astragaloside I | C₄₅H₇₂O₁₆ | 8.07 | [M+H]+ | 868.48204 | 869.4882 | -1.3 |
| 114 | Ginsenoside F2 | C₄₃H₇₄O₁₅ | 8.14 | [M-H]- | 784.49729 | 829.4937 | -2.2 |
| 115 | cis-Falcarindiol | C₁₇H₂₄O₂ | 8.18 | [M+H]+ | 260.17763 | 261.1858 | 3.4 |
| 116 | Ginsenoside Rg3 | C₄₃H₇₄O₁₅ | 8.25 | [M-H]- | 784.49729 | 829.4937 | -2.1 |
| 117 | (Z,Z,Z)-9,12,15-Octadecatrienoic acid | C₁₈H₃₀O₂ | 8.36 | [M+H]+ | 278.22458 | 279.2325 | 2.2 |
| 118 | Ligustilide | C₁₂H₁₄O₂ | 8.76 | [M+H]+ | 190.09938 | 191.1074 | 3.6 |
| 119 | 6-Aldehydo-isoophipogonone B | C₁₉H₁₆O₆ | 8.77 | [M+H]+ | 340.09469 | 341.1027 | 2.3 |
| 120 | Coniferyl ferulate | C₂₀H₂₀O₆ | 8.85 | [M+H]+ | 356.12599 | 357.1336 | 0.9 |
| 121 | Ruscogenin | C₂₇H₄₂O₄ | 8.87 | [M+H]+ | 430.30831 | 431.3152 | -0.8 |
| 122 | Ophiopogonin D | C₄₄H₇₀O₁₆ | 8.87 | [M+FA-H]- | 854.46639 | 899.4634 | -1.3 |
| 123 | Methyl ophiopogonanone A | C₁₉H₁₈O₆ | 8.92 | [M-H]- | 342.11034 | 341.1034 | 1 |
| 124 | Acetylastragaloside | C₄₇H₇₄O₁₇ | 9.06 | [M+FA-H]- | 910.4926 | 955.4889 | -2 |
| 125 | Methyl ophiopogonanone B | C₁₉H₂₀O₅ | 9.11 | [M-H]- | 328.13107 | 327.1236 | -0.6 |
| 126 | cis-9,cis-12-Linoleic acid | C₁₈H₃₂O₂ | 9.43 | [M-H]- | 280.24023 | 279.2325 | -1.8 |
| 127 | Ginsenoside Rg5 | C₄₂H₇₀O₁₂ | 9.55 | [M+FA-H]- | 766.48673 | 811.4848 | -0.1 |
| 128 | Levistolide-A | C₂₄H₂₈O₄ | 10.15 | [M+H]+ | 380.19876 | 381.2063 | 0.6 |
| 129 | Lecithin | C₄₂H₈₁NO₈P | 16.1 | [M+H]+ | 758.56998 | 759.5765 | -0.9 |
